# RIG-I Signaling via MAVS Is Dispensable for Survival in Lethal Influenza Infection *In Vivo*

**DOI:** 10.1155/2018/6808934

**Published:** 2018-11-08

**Authors:** Wenxin Wu, Xiaoqiu Wang, Wei Zhang, Lili Tian, J. Leland Booth, Elizabeth S. Duggan, Sunil More, Lin Liu, Mikhail Dozmorov, Jordan P. Metcalf

**Affiliations:** ^1^Pulmonary, Critical Care & Sleep Medicine, Department of Medicine, University of Oklahoma Health Sciences Center, Oklahoma City, Oklahoma, USA; ^2^Department of Pediatric Respiratory Medicine, Pubin Children Hospital, Shanghai Children Medical Center Affiliated to Shanghai Jiao Tong University School of Medicine, Shanghai 200092, China; ^3^The Lundberg-Kienlen Lung Biology and Toxicology Laboratory, Department of Physiological Sciences, Center for Veterinary Health Sciences, Oklahoma State University, Stillwater, Oklahoma, USA; ^4^Department of Biostatistics, Virginia Commonwealth University, Richmond, VA 23298, USA; ^5^Department of Microbiology and Immunology, University of Oklahoma Health Sciences Center, Oklahoma City, Oklahoma, USA; ^6^Veterans Affairs Medical Center, Oklahoma City, Oklahoma, USA

## Abstract

Retinoic acid-inducible gene I (RIG-I) is an important regulator of virus-induced antiviral interferons (IFNs) and proinflammatory cytokines. It requires interaction with an adaptor molecule, mitochondrial antiviral-signaling protein (MAVS), to activate downstream signaling pathways. To elucidate the mechanism(s) by which RIG-I-dependent recognition of IAV infection *in vivo* triggers innate immune responses, we infected mutant mice lacking RIG-I or MAVS with influenza A virus (IAV) and measured their innate immune responses. As has previously been demonstrated with isolated deletion of the virus recognition receptors TLR3, TLR7, and NOD2, RIG-I or MAVS knockout (KO) did not result in higher mortality and did not reduce IAV-induced cytokine responses in mice. Infected RIG-I KO animals displayed similar lung inflammation profiles as did WT mice, in terms of the protein concentration, total cell count, and inflammatory cell composition in the bronchoalveolar lavage fluid. RNA-Seq results demonstrated that all types of mice exhibited equivalent antiviral and inflammatory gene responses following IAV infection. Together, the results indicated that although RIG-I is important in innate cytokine responses *in vitro*, individual deletion of the genes encoding RIG-I or MAVS did not change survival or innate responses *in vivo* after IAV infection in mice.

## 1. Introduction

Infection with influenza A virus (IAV), a negative-sense single-strand RNA virus, is a major cause of morbidity and mortality. There are approximately 5 million clinical infections and 250,000–500,000 deaths resulting from yearly IAV epidemics around the globe, particularly in people over 65 years old who account for 90% of all influenza-associated deaths in the USA [1, 2].

Innate immunity is the first line of defense against virus infection that triggers the expression of interferon (IFN) and proinflammatory cytokines. Cells of the innate immune system detect viral infection largely through pattern recognition receptors (PRRs) present either on the cell surface or within distinct intracellular compartments. PRRs have the ability to distinguish self from nonself molecules. The innate immune system responds to influenza through three classes of PRRs. First, retinoic acid-inducible gene I (RIG-I) and melanoma differentiation-associated gene 5 (MDA5), widely expressed in various types of cells, such as myeloid dendritic cells (DC), macrophages, epithelial cells, and fibroblasts, detect intracellular ssRNAs and transcriptional intermediates of IAV [3, 4]. After recognition of virus, RIG-I or MDA5 binds to the downstream adaptor molecule, mitochondrial antiviral-signaling protein (MAVS), activating antiviral and proinflammatory signaling. Second, endosomal Toll-like receptors (TLRs) are also involved in IAV recognition. TLR3, a double-strand RNA sensor, is used by some epithelial cells and myeloid DC to detect the viral replicative intermediate dsRNA [5]. Plasmacytoid DC use TLR7 to recognize influenza genomic RNA upon release in late endosomes [6]. Finally, the nucleotide-binding domain and leucine-rich repeat-containing proteins (NLRP), including NLRP3 and nucleotide-binding oligomerization domain 2 (NOD2), may serve as intracellular mediators of IAV initiated host-cell signaling through the formation of a biochemical complex called the inflammasome in myeloid cells and airway epithelial cells [7–9].

The innate immune response triggered by PRR activation is essential for controlling viral infection. PRR receptors are the primary modulators of proinflammatory cytokine and chemokine production that activates leukocytes and recruits them to the site of infection, ideally optimizing immune responses and enhancing recovery [10]. However, excessive inflammation caused by an uncontrolled innate immune response is harmful to the host and contributes to mortality in IAV-infected patients [11]. The acute surge of cytokine release leads to an intense infiltration and activation of inflammatory cells, which is responsible for severe inflammation that exacerbates chronic lung diseases. Highly pathogenic IAV strains, including pandemic stains and avian influenza, are usually associated with excessive cytokine responses [12, 13].

RIG-I is essential for IFN induction during RNA virus infections of non-pDC cell types, and mice that are deficient in RIG-I-like receptor signaling pathways are extremely susceptible to other RNA viruses [14–16]. Our previous work using RIG-I transgenic mice showed that RIG-I overexpression in mice protects against cigarette smoke enhanced susceptibility of these animals to influenza infection [17]. Although PRRs are important in innate cytokine response *in vitro*, the deletion of genes encoding PRRs other than RIG-I does not worsen survival during IAV infection *in vivo*. In fact, mice deficient in TLR3 had an unexpected survival advantage during influenza infection perhaps due to significantly reduced inflammatory mediator induction in the animals [18]. Deletion of NOD2 did not change the survival rates of mice during lethal influenza infection [19].

In order to determine whether RIG-I signaling was important for survival and IAV-induced cytokine responses in mice, we infected mutant mice lacking RIG-I or MAVS with IAV, measured their innate immune responses, including IFN and proinflammatory cytokine induction, and determined their mortality. The mechanism(s) by which RIG-I-dependent recognition of IAV infection *in vivo* triggers innate immune responses was also evaluated.

## 2. Results

### 2.1. RIG-I Is Not Required for Survival in Lethal IAV Infection

**Figure 1:**
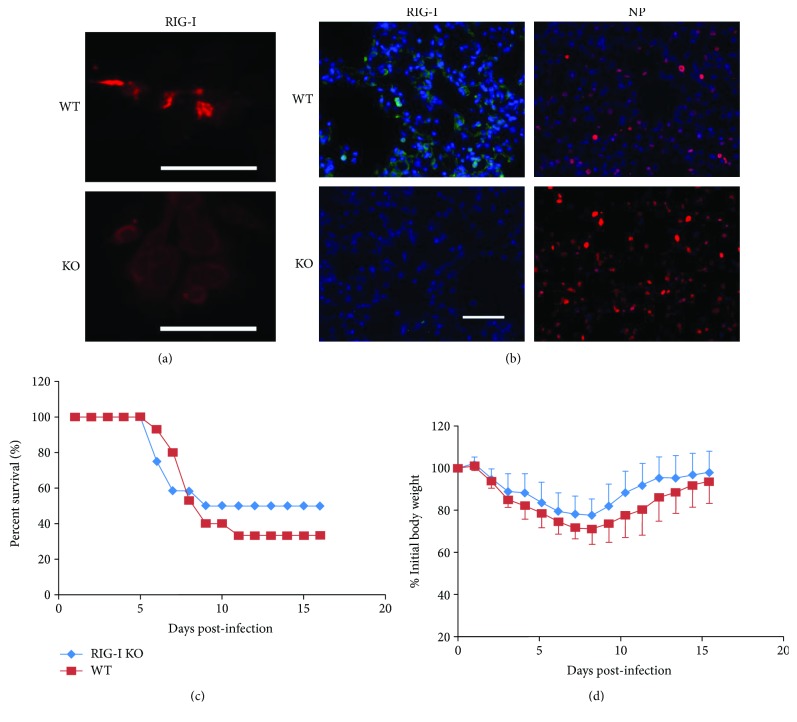
RIG-I is dispensable for survival and weight loss during influenza infection. (a) Wild-type (WT), but not RIG-I KO, mice express RIG-I in isolated type II alveolar epithelial cells (AEC). AEC II were infected with IAV PR8 at an MOI of 6 and incubated for an additional 24 h to stimulate RIG-I production. The cells were processed for immunohistochemistry for detection of RIG-I (red). Scale bars = 50 *μ*m. (b) WT, but not RIG-I KO, mice infected with IAV express RIG-I in the lung. Immunohistochemical staining of RIG-I and IAV nucleoprotein (NP) in WT and RIG-I KO mice. Mice were intranasally infected with 300 pfu IAV PR8 or mock infected with PBS. Mouse lungs were processed for immunohistochemistry for detection of RIG-I protein (green) or IAV NP (red). The bar represents 100 *μ*m. (c and d) RIG-I KO and littermate WT mice were intranasally inoculated with IAV at 1000 pfu/mouse. Mortality (c) and body weights (d) were monitored daily. Body weight data were normalized to each mouse's starting body weight. Data are expressed as mean ± standard deviation (*n* = 13 for RIG-I KO mice; *n* = 15 for WT mice).

RIG-I^−/−^ mice in a C57BL/6 background were prepared as described in Materials and Methods and, as with all other mouse strains used, were genotyped and bred under pathogen-free conditions in the animal facility at the University of Oklahoma Health Sciences Center. To confirm RIG-I disruption, we isolated lung AEC II from RIG-I knockout (KO) and wild-type (WT) mice. Isolated cells were cultured for 2 days, infected with IAV for 24 h, and stained for RIG-I. IAV-infected WT AEC express high levels of RIG-I while infected RIG-I KO AEC do not express RIG-I (Figure 1(a)). We also confirmed RIG-I KO in mouse lung by immunostaining. Mice were infected with IAV and sacrificed after 6 days. Lungs were processed for immunohistochemistry for detection of IAV nucleoprotein (NP) and RIG-I. PBS mock control KO and WT mouse lungs had minimal immunofluorescence when stained for RIG-I. As expected, RIG-I was highly induced in lungs from IAV-infected WT mice. Viral NP expression was detected in lungs from both WT and KO animals when infected with IAV (Figure 1(b)). The results demonstrate that virus replicated in mouse lungs after IAV infection with concurrent induction of RIG-I. The data demonstrate that RIG-I protein expression is deficient in RIG-I KO AEC II and in RIG-I KO mouse lung, even when infected with IAV.

In order to determine the *in vivo* role of RIG-I in survival during severe IAV infection, we inoculated RIG-I KO and littermate WT animals with a lethal dose of virus (1000 pfu). Unexpectedly, we found that RIG-I KO mice had no significant difference in survival after IAV challenge as compared to similarly exposed WT mice by Kaplan-Meier survival analysis (*p* = 0.696, *n* = 13 and 15, respectively, Figure 1(c)). Weight loss was significant in both infected groups and reached a nadir at 7 days after infection though it did not differ significantly between the groups (Figure 1(d)).

### 2.2. Inflammatory Responses in the Lung Are Induced during IAV Infection Even in the Absence of RIG-I

To investigate the role of RIG-I in the inflammatory response to IAV, RIG-I KO and WT C57BL/6 mice were intranasally infected with 300 pfu IAV PR8. The mock group was sham infected by inoculation with a single dose of an equal volume of PBS. Animals were sacrificed at 2, 4, and 6 days after infection, and bronchoalveolar lavage fluids (BALF) were collected to assess cellular infiltration and mediator content in the airspaces.

**Figure 2:**
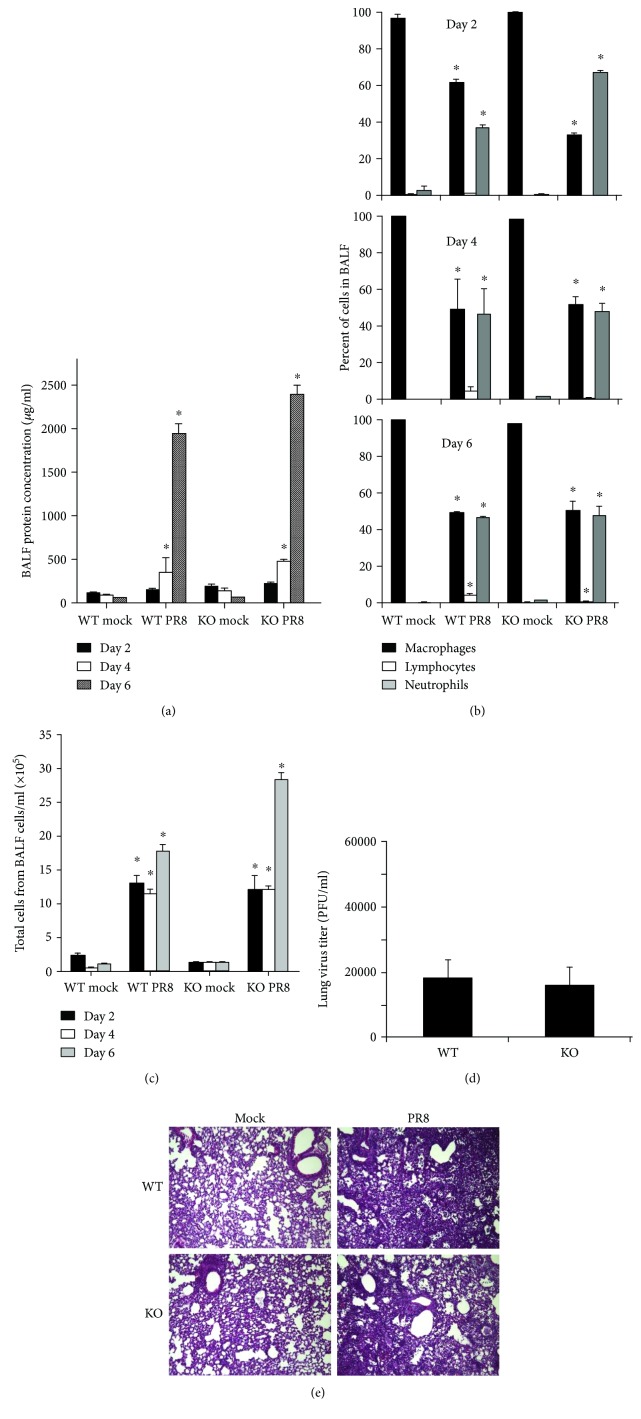
Inflammatory profile in the bronchoalveolar lavage fluid (BALF) and virus titer in the lung. WT and RIG-I KO mice were intranasally infected with 300 pfu of the IAV PR8 or mock infected with PBS. BALF was harvested at the indicated time points after infection. Total protein levels (a), immune cell differential (b), and total cells (c) in BALF were determined. Cytospins of the cells were prepared using a Cytopro Cytocentrifuge and stained with Diff-Quik. Differential counts were manually determined using the morphology of more than 400 cells/sample from 2 slides/mouse. Lung tissue viral titers were determined at 6 days postinfection by plaque assay on MDCK cells (d). Data are expressed as means ± SEM (*n* ≥ 3/group). ^∗^ denotes significant difference compared to the corresponding mock infected groups (*p* < 0.05). (e) Mouse lung tissue pathology after IAV infection. Mice were intranasally infected with 300 pfu of IAV PR8 strain. Samples were harvested after 6 days. Lung tissue sections prepared from the infected mice were fixed, processed, and stained with H&E. The lungs of 3 mice from each treatment group were processed for histology, and results shown were typical for the group (200x magnification).

BAL is the most common manner to sample the components of the epithelial lining fluid and to determine the amount of total protein, an index of transudation from the vascular compartment into the lungs, and reflects lung injury. We found no significant difference in BAL protein levels in both infected mouse groups at all time points (Figure 2(a)).

In terms of total inflammatory cells, IAV inoculation not only caused a significant increase in the total viable leukocytes in BALF from day 2 but also significantly increased the percentage of neutrophils in BALF in all infected groups (Figures 2(b) and 2(c)). However, RIG-I KO did not significantly alter the composition of the inflammatory cell population during viral infection. At day 6 after infection, RIG-I KO mice appeared to have more inflammatory cell infiltration into BALF than did WT mice, but the difference was not statistically significant. Thus, the total viable cell numbers in BALF were similar in both mouse groups at all time points. Plaque assays of whole lung showed that viral titers were equally elevated in WT and KO mice after 6 days of infection (Figure 2(d)). Thus, as for mortality, the viral burden in the lung was not altered by RIG-I KO.

Examination of histopathology revealed that IAV-infected lungs in both types of mice showed typical viral pneumonia with interstitial edema and inflammatory infiltration, as well as necrotizing bronchitis and bronchiolitis. IAV infection resulted in the expected neutrophilic alveolar infiltrate with some lymphocytes. However, we found little difference in terms of the severity of inflammation in RIG-I KO and WT mice (Figure 2(e)).

### 2.3. RIG-I Deficiency Does Not Alter Antiviral Interferon and Inflammatory Cytokine Responses to Influenza Infection

**Figure 3:**
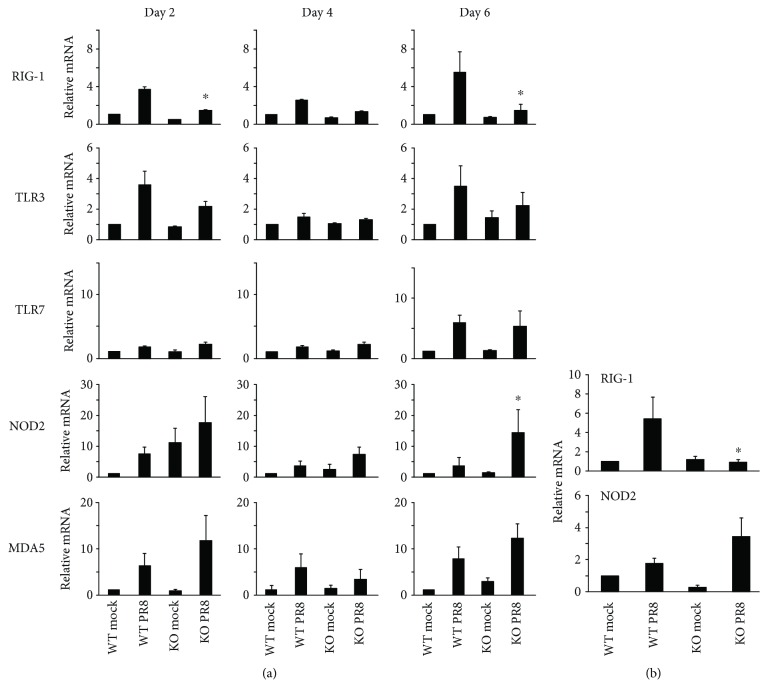
Virus PRR mRNA induction in RIG-I KO mice during IAV infection. (a) mRNA induction in mouse lung. Mice were infected with 300 pfu of the IAV PR8 strain. Mock-treated mice were inoculated with PBS. At the indicated time points, the mice were sacrificed and lung tissues were collected for RNA preparation. (b) mRNA induction in primary ear fibroblasts infected with IAV. Primary ear fibroblasts isolated from RIG-I WT and KO mice were infected with IAV PR8 at an MOI of 1. After 24 h, cells were collected for RNA extraction. The mRNA levels were assessed by qRT-PCR and normalized by *β*-actin. Data are expressed as means ± SEM of fold increase over the WT mock group (*n* ≥ 3 per group). For clarity, we only show significant differences (^∗^ *p* < 0.05) between the PR8-infected RIG-I KO group and the PR8-infected WT group. *p* value was calculated from the ΔΔCt values from different experimental groups.

In order to determine how mice survive IAV infection in the absence of a major antiviral sensor, we assessed PRR and cytokine expression during infection of both mouse strains. Mice were inoculated intranasally with a single, nonlethal dose of the IAV PR8 strain (300 pfu). Lung tissues and BALF were collected at 2, 4, and 6 days after infection, and PRR and cytokine expression was determined by qRT-PCR or multiplex immunoassay, respectively. All tested PRR mRNAs were induced by virus in lungs from WT mice at 2 days after PR8 infection (Figure 3(a)). Specifically, pulmonary expression of RIG-I was markedly upregulated following influenza infection in WT mice. As expected, there was no RIG-I expression in RIG-I KO mice with PR8 infection. Remarkably, we found that NOD2 mRNA expression was much greater in RIG-I KO mouse lung as compared with WT mice, especially at day 6 after infection. PR8 induction of NOD2 mRNA in RIG-I KO mice (15-fold) was significantly greater than in WT mice (2-fold, Figure 3(a)). This suggests compensatory NOD2 overexpression in the absence of RIG-I. To confirm this *in vitro*, we isolated fibroblasts from the ears of RIG-I KO and WT mice. NOD2 mRNA induction by IAV was greater in cells from KO mice than in cells from WT mice (Figure 3(b)).

**Figure 4:**
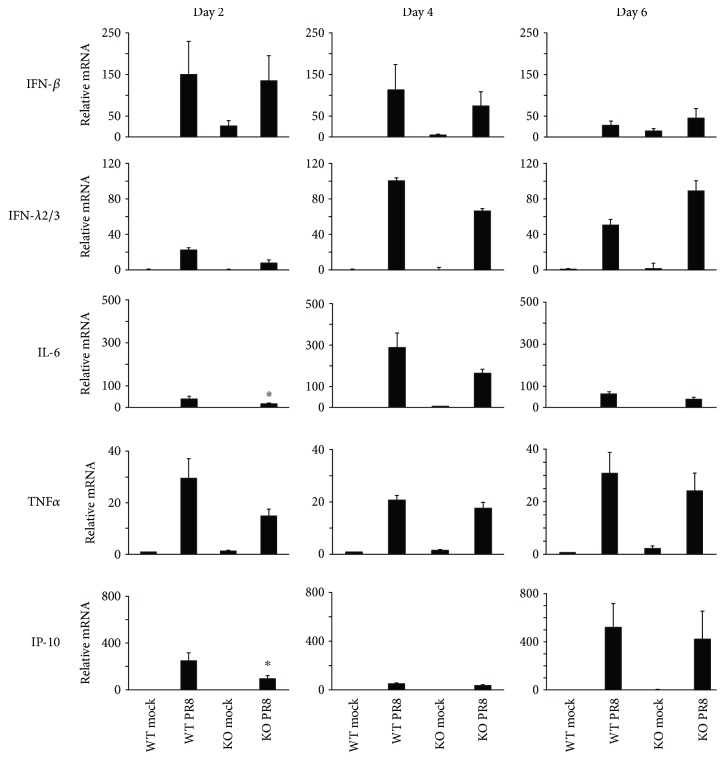
Antiviral and proinflammatory cytokine mRNA induction during IAV infection. Mice were intranasally infected with 300 pfu of the IAV PR8 strain. Mock-treated mice were inoculated with PBS. At the indicated time points, the mice were sacrificed and lung tissues were collected for RNA extraction. IFN and cytokine mRNA levels were assessed by qRT-PCR and normalized to *β*-actin. Data are expressed as means ± SEM of fold increase (*n* ≥ 3 per group). ^∗^ denotes significant difference compared to the PR8 infected WT group, *p* < 0.05. *p* value was calculated from the ΔΔCt values from different experimental groups.

**Figure 5:**
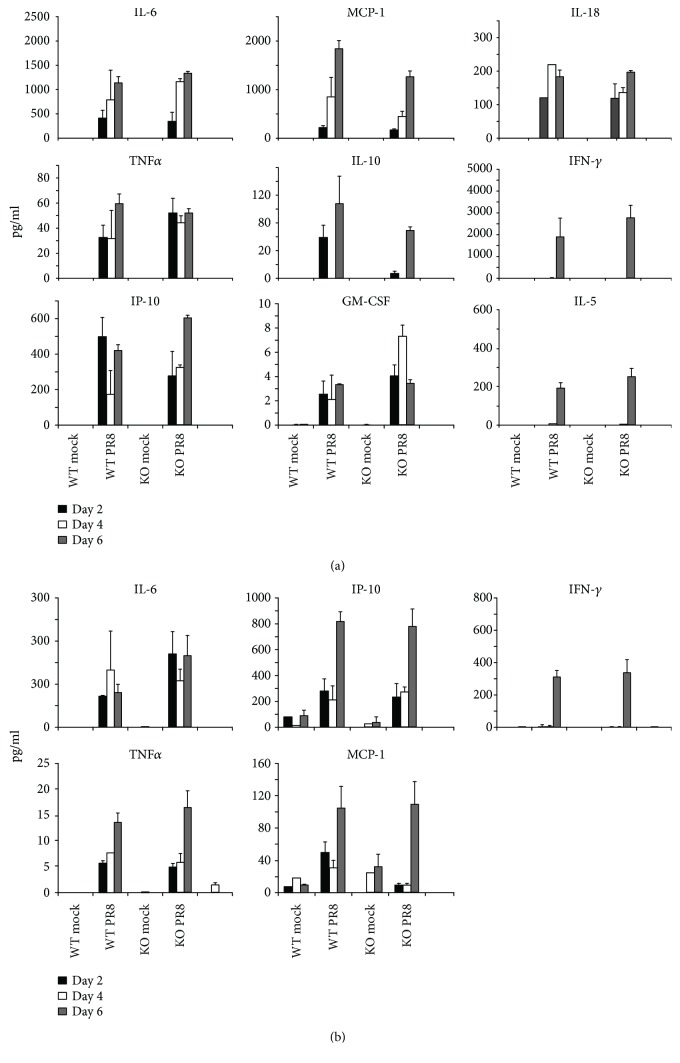
Antiviral and proinflammatory cytokine protein levels in BALF and serum in RIG-I KO and WT mice during IAV infection. Mice were intranasally infected with 300 pfu of the IAV PR8 strain. (a) BALF and (b) serum were harvested at the indicated time points after infection. Mock-treated mice were inoculated with PBS. Antiviral and proinflammatory cytokine protein levels were determined by multiplex immunoassay. Data are expressed as mean ± SEM (*n* ≥ 3 per group).

We also measured IFN and cytokine mRNA induction in response to IAV in these animals. The IFN-*β* and *λ*2/3 response to flu infection was similar in RIG-I KO and WT mice. Consistent with the inflammatory cell profile in BALF, mRNA expression of the proinflammatory cytokines IL-6, TNF*α*, and IP-10 was highly and similarly induced in both genotypes during IAV infection (Figure 4). The data suggest that RIG-I is not required for the innate antiviral and proinflammatory cytokine response to IAV in mice. Finally, to confirm that cytokine induction was reflected at the level of translation, we measured cytokine proteins in BALF and blood cytokine levels from mice exposed to IAV in both genotypes using multiplex immunoassay (Figure 5). The protein levels of IL-6, MCP-1, TNF*α*, IP-10, and IFN-*γ* in serum showed similar patterns to those seen in BALF.

### 2.4. MAVS Is Dispensable for Survival in IAV Infection *In Vivo*

**Figure 6:**
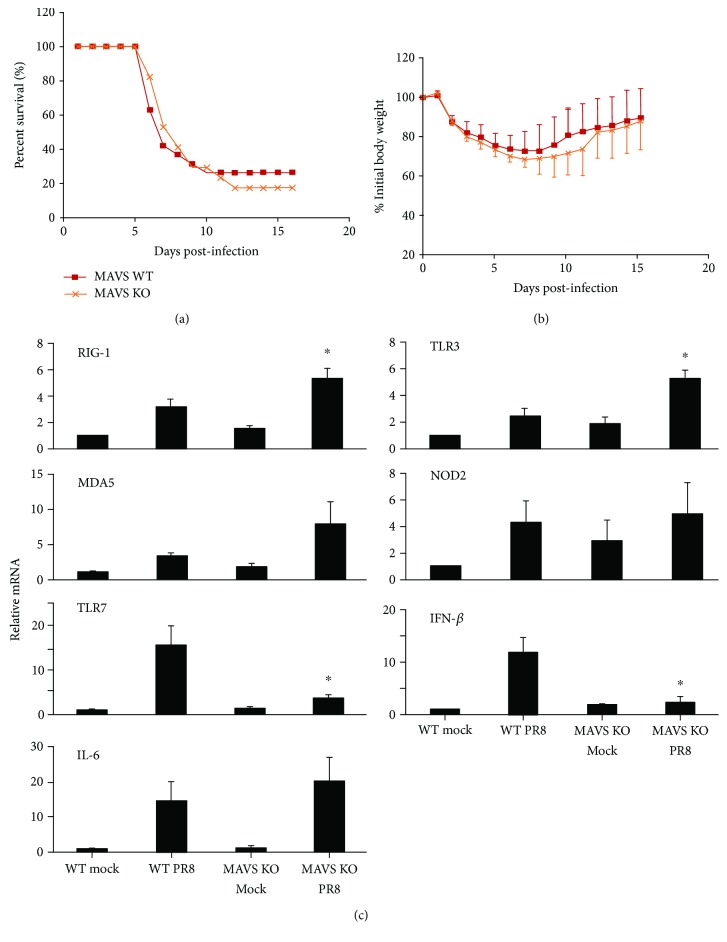
MAVS is dispensable for survival and cytokine induction during influenza infection. MAVS KO and littermate WT mice were intranasally inoculated with IAV at 1000 pfu/mouse. Mortality (a) and body weights (b) were monitored daily. Body weight data were normalized to each mouse's starting body weight. Data are expressed as mean ± standard deviation (*n* = 18 for MAVS KO mice; *n* = 19 for WT mice). (c) PRR and cytokine mRNA induction in mouse lung. Mice were infected with 300 pfu of the IAV PR8 strain. Mock-treated mice were inoculated with PBS. At day 6 postinfection, the mice were sacrificed and lung tissues were collected for RNA preparation. The mRNA levels were assessed by qRT-PCR and normalized *β*-actin. Data are expressed as mean ± SEM of fold increase (*n* ≥ 3 per group). For clarity, we only show significant differences (^∗^ *p* < 0.05) between the PR8-infected MAVS KO group and the PR8-infected WT group. *p* value was calculated from the ΔΔCt values from different experimental groups.

In order to further examine the role of RIG-I pathway activation in the response to IAV infection, we next investigated whether depletion of MAVS, the RIG-I adaptor, affects the survival rate during IAV infection. We inoculated MAVS KO and littermate WT animals with a lethal dose of virus (1000 pfu). As we found for RIG-I KO, MAVS KO had no significant effect on survival after IAV challenge, with approximately 18% and 23% survival at 16 days in MAVS KO and WT mice (*n* = 18 and 19, respectively, Figure 6(a)). Weight loss was significant in both infected groups and reached a nadir at 7 days after infection (Figure 6(b)). The body weight data agreed with the survival data in that there was no significant difference in weight loss between IAV-infected MAVS KO and WT mice (approximately 31% vs. 28% loss for MAVS KO and their littermate WT at day 7).

We also compared PRR and cytokine expression during IAV infection of both mouse strains. Mice were inoculated intranasally with a single, nonlethal dose of the IAV PR8 strain (300 pfu). Lung tissues were collected at 6 days after infection, and PRR and cytokine expression was determined by qRT-PCR. Again, all tested PRR mRNAs were induced by virus in lungs from WT mice after PR8 infection (Figure 6(c)). Lung expression of RIG-I and MDA5 was modestly upregulated following influenza infection in MAVS KO mice as compared with WT mice, suggesting that there was attempted compensation for MAVS KO by overexpression of RIG-I and MDA5. There was also a markedly compensatory increase in TLR3 expression in MAVS KO mice. Surprisingly, we found that TLR7 mRNA expression was significantly lower in MAVS KO mice as compared with WT mice. To assess downstream effects of PRR signaling, we measured IFN-*β* and IL-6 cytokine mRNA induction in response to IAV in these animals. The IL-6 response to flu infection was similar in RIG-I KO and WT mice, but IFN-*β* mRNA expression was significantly reduced in MAVS KO mice as compared to that seen in WT mice. The data suggest that IFN-*β* expression is affected in the lung at day 6 while proinflammatory cytokine response is the same in both types of mice during IAV infection.

### 2.5. Interferon and Inflammatory Response Genes Are Induced during IAV Infection Even in the Absence of RIG-I or MAVS

To better understand the effect of modulation of RIG-I signaling on global gene expression, we used high-throughput RNA sequencing (RNA-Seq) technology, a powerful way to profile the transcriptome with great efficiency and higher accuracy. RNA-Seq was performed on mRNA derived from WT, RIG-I KO, and MAVS KO mouse lungs at 6 days postinfection. Using the 43,304 annotated genes in the mouse genome database, gene expression was quantified and compared between the mock and the virus-infected groups, and differentially expressed genes (DEGs) were identified using a false discovery rate (FDR) of 0.1.

**Figure 7:**
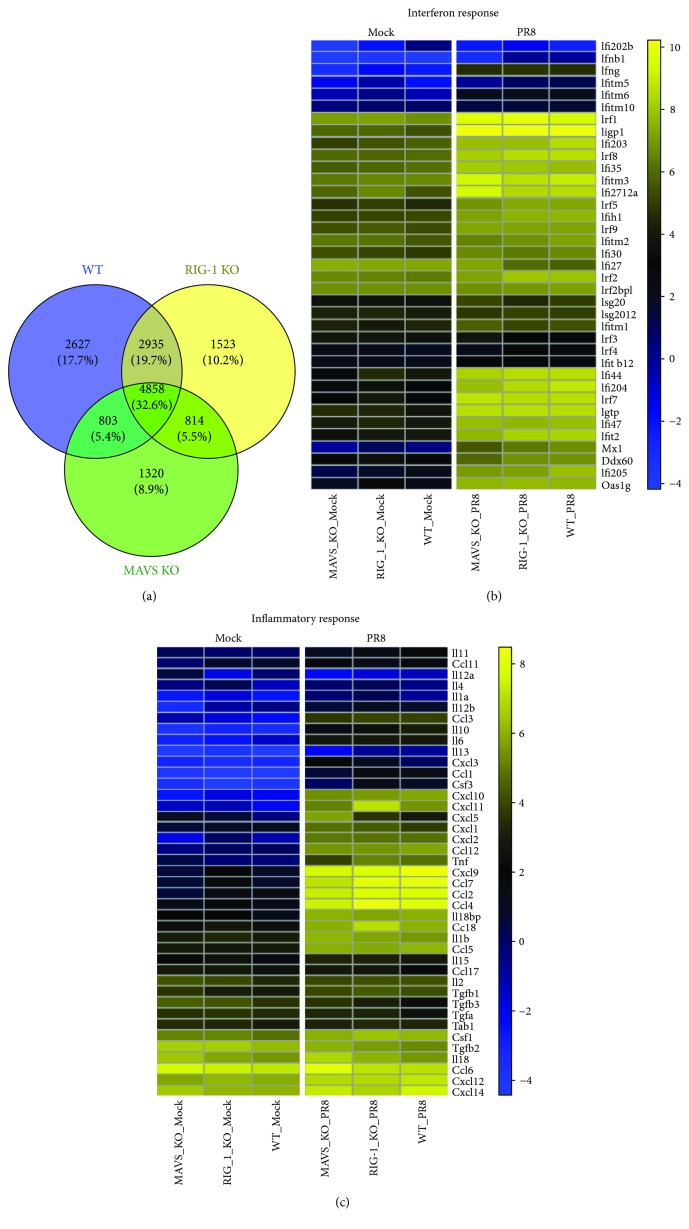
The absence of RIG-I or MAVS does not inhibit induction of interferon or generation of an inflammatory response during IAV infection. WT, RIG-I KO, and MAVS KO mice were mock-infected or infected with the IAV PR8 strain (300 PFU/mouse). At day 6 postinfection, lung tissue was collected, and total RNA was isolated and analyzed for RNA-Seq using 3′ inTAG next-generation sequencing (*n* = 6). (a) Venn diagram showing the distribution of shared differentially expressed genes during IAV infection. Heat map comparing the genes of interferon (b) and inflammatory responses (c). The log2 counts per million for the corresponding gene sets were averaged for each group and clustered as heat maps using the Euclidean distance and Ward clustering metric. The yellow/blue gradient indicates high/low gene expression, respectively.

All three types of mice shared 32.6% of total DEGs during infection (Figure 7(a)). The DEGs that distinctively changed in each strain only accounted for 17.7% (WT), 10.2% (RIG-I KO), and 8.9% (MAVS KO). To examine the biological roles, a Gene Ontology (GO) enrichment analysis was applied to the DEGs (Figure S1). Notably, the DEGs were mostly enriched in the regulation of innate immune reactions, such as the defense response, the antiviral response, and the inflammatory response. Nine out of 12 biological processes overlap each other between WT and RIG-I or MAVS KO mice during IAV infection.

Two key innate immune responses that occur during IAV infection and impact survival are interferon responses and inflammatory responses. Therefore, we focused on DEGs in these categories with an FDR of 0.1 that were upregulated by IAV exposure in the WT and KO mouse groups. The log_2_ counts per million for the corresponding gene sets were averaged for each group and clustered as heat maps using Euclidean distance and the Ward clustering metric. The yellow/blue gradient indicates high/low gene expression, respectively. In the first set (Figure 7(b)) are genes related to the interferon response genes, including Irf1, Ifi44, Irf7, and Oas1g. The second gene set (Figure 7(c)) included genes related to inflammatory responses, such as Ccl5, Il6, Il1b, and Tnf. The gene expression data shows that IFN response and inflammatory genes were induced by IAV in the absence of RIG-I and MAVS. As demonstrated in Figure 6, RNA-Seq also showed that IAV infection induced IFNb1 3-fold over mock in MAVS KO mice. The induction by IAV was much less than that seen in WT mice. In contrast, MAVS KO did not affect induction of downstream IFN-stimulated genes (ISGs, Ifi44, Ifi204, Ifi47, Ifit2, Ifi205, etc.) by IAV. As downstream genes are similarly activated in KO mice despite differential IFN-*β* induction, it is likely that other IFNs or cytokines might compensate for IFN-*β* in the mouse lung [20].

## 3. Discussion

The critical role of the RIG-I-MAVS-dependent pathway in recognizing IAV infection and controlling pathogenesis has been established and implies an essential role for RIG-I in immunity against IAV [3, 4, 21]. To further investigate the impact of RIG-I specifically *in vivo*, it would be ideal to examine IAV pneumonia in RIG-I-deficient mice. Two groups previously reported that RIG-I-deficient C57BL/6 mice had developmental defects and have high mortality during embryogenesis [3] or within 3 weeks after birth as a result of extensive hepatocellular apoptosis [22]. A successful approach to generate viable RIG-I^−/−^ mice has been to generate them in a complex background involving 129Sv, C57BL/6, and ICR mice and repeatedly backcross the mice into C57BL/6. We obtained early generation founders of these mice (a kind gift from Dr. Michael Gale, University of Washington) and backcrossed them into a C57BL/6 background to the F4 generation. Similar viable mice containing MDA5^−/−^ in addition to RIG-I^−/−^ in this background have been developed by his group in this manner [23]. The RIG-I^−/−^ mouse line developed in our laboratory described herein has an approximately 94% C57BL/6 genetic background, as determined by microsatellite DNA analysis (see Materials and Methods).

Here, our work showed that RIG-I^−/−^ mice had similar survival and body weight loss compared to WT mice following IAV infection. Infected RIG-I^−/−^ animals displayed a similar pattern of lung inflammation as did WT mice. RNA-Seq results showed that RIG-I KO, MAVS KO, and WT mice exhibited similar induction of antiviral and inflammatory genes following intranasal challenge with IAV. The data suggest that RIG-I's role in the recognition and inhibition of IAV can be replaced by other PRRs in mice. Investigations by other groups demonstrating similar inflammatory responses to IAV in the absence of other PRRs provide additional evidence for the existence of compensatory or complementary *in vivo* mechanisms of PRR induction. For example, TLR7 deficiency does not alter survival and viral clearance following IAV infection but exacerbates body weight loss [24]. Also, lung mRNA expression of IFNs and chemokines from mice infected with IAV is not affected by isolated knockout of the MyD88 or MAVS pathway. This suggests that either the MyD88 or MAVS signaling pathway is sufficient for initial antiviral responses to IAV *in vivo* [25]. Our group has also reported that neither RIG-I nor TLR3 siRNA alone completely blocked IFN induction in human lung epithelial cells. Only double knockdown of RIG-I and TLR3 completely inhibited IFN induction by influenza. This shows that signaling compensation of RIG-I for TLR3, or vice versa, preserving IFN induction by IAV occurs in human lung [26]. Our results combined with other reports strongly suggest that innate immune responses to IAV are not regulated by a single receptor or intracellular signaling pathway. In fact, the response appears to be a well-orchestrated process, which involves multiple complementary PRRs and signaling pathways in different cells and tissues of the body.

Notably, we found that NOD2 or TLR3 mRNA expression was greatly increased in IAV-infected RIG-I or MAVS KO mice, respectively. Thus, activation of NOD2 following IAV infection of mice could compensate for the absence of functional RIG-I. Morosky et al. have shown that RIG-I and NOD2 not only are colocalized to cellular ruffles and cell-cell junctions but also interact directly [27]. Moreover, RIG-I negatively regulates ligand-induced NF-*κ*B signaling mediated by NOD2, and NOD2 negatively regulates type I IFN induction by RIG-I. At the cellular level, it seems likely that RIG-I expression negatively regulates NOD2 signaling and expression, and *vice versa*. In the absence of inhibition of NOD2 signaling by RIG-I in RIG-I^−/−^ mice, unrestrained NOD2 might optimize innate immune responses to viral infections and improve survival. Recently, RNAi screening has implicated the other RIG-I-like receptor, MDA5, as a significant contributor to the cellular defense against IAV [28].

Our current model is that RIG-I serves as the primary PRR for IAV-mediated cytokine induction in the primary IAV infection sites, lung epithelia and macrophages, and that TLR3, NOD2, MDA5, and TLR7 in epithelia and other immune cells serve as important alternate PRRs for generating an innate response to IAV. Our findings show that host recognition of IAV by PRR *in vivo* and initiation of innate immunity are more complex than currently appreciated in that two or three pathways compensate for one another in upregulating antiviral responses. There might be extensive cooperative and/or competitive interactions among different PRRs that support and regulate antiviral sensing and induction of innate immune responses. Our previous publication demonstrated that RIG-I overexpression in the lung improves survival of cigarette smoke-exposed mice during IAV infection [17]. This suggests that, although solitary PRR deficiency might be dispensable in innate response to IAV, overexpression of one major PRR is sufficient to restore the innate response to IAV infection in an immunosuppressed cohort.

Together, our results demonstrate that RIG-I is dispensable for the innate cytokine response to IAV. These results, together with our earlier report showing that overexpression of RIG-I in the lung improves survival during viral infection in smoke-exposed mice, provide new insight into the mechanisms on how the host immune system maintains homeostasis during influenza infections. More studies are required to elucidate the underlying mechanisms of control of viral infection and virus-mediated excessive inflammation by the innate immune response.

## 4. Materials and Methods

### 4.1. Ethics Statement

The Institutional Animal Care and Use Committee (IACUC) of the University of Oklahoma Health Sciences Center approved all of the protocols for the animal experiments (protocol number: 17-106-HI). The facility where this research was conducted is accredited by AAALAC. The facility operates according to the Guide for the Care and Use of Laboratory Animals and the requirements of the Animal Welfare Act and Regulations and the Public Health Service Policy on Humane Care and Use of Laboratory Animals. All procedures were performed by personnel trained in the techniques according to IACUC guidelines. All invasive clinical procedures were performed while animals were anesthetized.

### 4.2. Preparation of Influenza Virus Stock and Plaque Assays

H1N1 influenza virus, A/PR/34/8 (PR8), was passaged in Madin-Darby canine kidney (MDCK, ATCC, #CCL-34™, Manassas, VA) cells. Virus was grown in MDCK cells in DMEM/F12 with ITS+ (BD Biosciences, Franklin Lakes, NJ) exposed to trypsin, harvested at 72 hours postinfection, and titered by plaque assay in MDCK cells. There was no detectable endotoxin in the final viral preparations used in the experiments as determined by limulus amebocyte lysate assay (Cambrex, Walkersville, MD). The lower limit of detection of this assay is 0.1 EU/ml or approximately 20 pg/ml LPS. For determination of viral titers in infected mice, whole mouse lungs were collected and homogenized in 1 ml of ice-cold PBS. Solid debris was pelleted by centrifugation, and viral titer was determined using a standard plaque assay on MDCK cells.

### 4.3. Animals

Specific pathogen-free MAVS KO mice with mixed C57BL/6 and 129SvEv genetic background were purchased from the Jackson Laboratory (Bar Harbor, ME). RIG-I^−/−^ mice were generated by Dr. S. Akira's group on a mixed ICR × 129Sv × C57BL/6 genetic background. The RIG-I^−/−^ mice were backcrossed into a C57BL/6 background through the F4 generation and had no developmental defects. The F4 mice were 90–94% C57BL/6 background as determined by JAX using genome scanning (Table 1).

All mice were genotyped and bred under pathogen-free conditions in the animal facility at the University of Oklahoma Health Sciences Center. Mice were housed at 20°C on a 12-hour light/dark cycle in sterile microisolator cages and fed *ad libitum* with sterile chow and water.

### 4.4. Influenza Virus Infection

IAV infection was performed under isoflurane anesthesia. IAV PR8 stock was diluted in PBS to make lethal and sublethal doses of virus. These virus doses (50 *μ*L solution) were administered by intranasal instillation as the animal was held in a vertical position while being sedated. Control animals received PBS. Mice were monitored daily for 16 days for clinical symptoms (shaking, tiredness, and piloerection), and their weight was recorded daily.

### 4.5. Bronchoalveolar Lavage (BAL)

Mice were sacrificed using isoflurane. BAL was performed using a closed thorax technique by exposing the trachea, nicking the bottom of the larynx, and inserting a 3/4-inch 22-gauge cannula into the proximal trachea. The proximal end of the trachea was tied off, and 0.6 ml of sterile PBS was gently introduced into the lungs and recovered. This was repeated 3 times for a total volume of 1.8 ml. Return volume varied by <10% between samples. BALF was centrifuged to remove cells. Cells obtained were placed on slides for determination of cell populations using a Cytopro Cytocentrifuge (Wescor, Logan, UT) and stained with Diff-Quik (Dade Behring, Newark, DE). Differential counts were made with ≥400 cells/sample from 2 slides/mouse. The BALF was pooled and frozen.

### 4.6. Multiplex Immunoassay

Cytokine protein levels in the BALF and serum were determined by multiplex immunoassay (Affymetrix, Santa Clara, CA). The assay was run on a Bio-Plex 200 multiplex system (Bio-Rad, Hercules, CA).

### 4.7. Measurement of mRNA Expression by Quantitative Real-Time PCR (qRT-PCR)

Total RNA from lung was extracted using a modified TRIzol (Invitrogen, Carlsbad, CA) protocol and spectrophometrically quantitated. The integrity of RNA was verified by formaldehyde agarose gel electrophoresis. Equal amounts (1 *μ*g) of RNA from each sample were reverse-transcripted into cDNA with oligo (dT) SuperScript II First-Strand Synthesis System for RT-PCR (Invitrogen, Carlsbad, CA). Gene-specific primers for mouse PRRs, cytokines, and the *β*-actin housekeeping genes were used. The primers' sequences were the same as in our earlier publication [17]. qRT-PCR was performed using 100 ng sample RNA and SYBR Green (Quanta Biosciences, Gaithersburg, MD) in a Bio-Rad CFX96™ Touch Real-Time PCR Detection System. Results were calculated and graphed from the ∆CT of target gene and normalizer, *β*-actin.

### 4.8. Isolation of Primary Ear Fibroblast from Mice

Mice were euthanized using isoflurane. The left and right pinna were removed and minced using sterile scissors in growth media (DMEM, 10% FBS, 1x nonessential amino acids [Gibco, Cat. #11140-050], 1x Pen-Strep from Gibco) supplied with collagenase (4 mg/ml; Worthington, Lakewood, NJ) and protease (Dispase II, 4 mg/ml; Roche) to enhance cell extraction. The resultant suspension was cultured overnight in a 37°C incubator. A single-cell suspension of this preparation was obtained by passage through a cell strainer twice to remove remaining debris. Cells were pelleted by centrifugation at 1020 × g for 5 minutes. Cells were pooled from both pinna and resuspended in 5 ml of growth media and brought up to 25 ml with growth media. Cells were plated on a round 150 mm plate and allowed to adhere overnight in a 37°C incubator. Following the overnight incubation, media were replaced and cells were grown to confluence prior to infection with IAV.

### 4.9. Histological and Immunohistochemical Analysis of Mouse Lung Tissue and Alveolar Epithelial Cells (AEC) II

At day 6 after IAV infection, mice were sacrificed and lungs were fixed in 4% paraformaldehyde in PBS at room temperature for 30 minutes and were then embedded in paraffin. Fixed tissue was hematoxylin and eosin- (H&E-) stained to assess inflammation and fibrosis. Sections (3–5 *μ*m) were mounted on glass slides and immunoprobed with a rabbit anti-mouse polyclonal antibody for RIG-I (Abcam) or an anti-NP polyclonal antibody [29]. AEC II were purified and plated on collagen-coated glass slides and cultured [30]. After 1 day, the cells were infected with IAV PR8 at an MOI of 1 and incubated for an additional 24 h to stimulate RIG-I production. The cells were probed with a rabbit anti-mouse polyclonal antibody for RIG-I (Santa Cruz Biotechnology). Nuclei were stained with DAPI (blue). After washing, the sections were probed with a donkey anti-rabbit secondary antibody conjugated to Alexa Fluor 546 (BD/Molecular Probes). Transmitted light and fluorescent microscopy images were obtained using an Olympus BX51 microscope running cellSens imaging software (Olympus, Center Valley, PA).

### 4.10. RNA-Seq Analysis

Mouse lung total RNA was collected as described earlier. Library preparation and sequencing were conducted using 3′ inTAG next-generation sequencing by the Clinical Genomics Center Core Facility of the Oklahoma Medical Research Foundation (Oklahoma City, OK). Differential gene expression for day 6 post-IAV infection was determined relative to mock inoculated mice.

All sequencing reads were quality-controlled using FastQC v0.11.2. Illumina adapters were trimmed using Cutadapt v1.9.dev2; replicates were merged and aligned with their reference genome (UCSC mouse genome build mm10) using Subread-align v1.4.6-p4. The BAM files from alignment were processed using featureCounts v1.4.6-p4 to obtain the counts per gene in all samples. Mus_musculus.GRCm38.83.gtf gene definition file was used. The differential expression analysis was performed using edgeR v3.18.1. Genes having counts per million less than 2 in all samples were excluded. Differentially expressed genes were defined using *p* value <0.01 and FDR-corrected *p* value <0.1 cutoffs. All bioinformatics analyses were conducted in the R/Bioconductor computing environment v3.4.0.

### 4.11. Statistical Analysis

Where applicable, for data other than that related to RNA-Seq, the data was expressed as mean ± standard error of the mean (SEM). Statistical significance was determined by one-way ANOVA with Student-Newman-Keuls post hoc correction for multiple comparisons. Significance was considered as *p* < 0.05. For RT-PCR results, the *p* value was calculated from the ΔΔCt values from different experimental groups.

## Figures and Tables

**Table 1:** Genome scanning to determine C57BL/6 background of the RIG-I^−/−^ mice.

Sample	129S1/SvImJ	C57BL/6J
1	8.84%	91.16%
2	9.52%	90.48%
3	6.46%	93.54%
4	7.19%	92.81%
5	5.10%	94.90%
6	6.42%	93.58%
7	8.50%	91.50%
8	7.82%	92.18%
9	5.44%	94.56%
10	6.51%	93.49%
B6J	0.00%	100.00%
129S	100.00%	0.00%
Heterozygous	50.00%	50.00%

## Data Availability

All RNA-Seq files are available from the GEO database (accession number GSE114232), https://www.ncbi.nlm.nih.gov/geo/query/acc.cgi?acc=GSE114232.

## Supplementary Materials

Supplementary Materials Figure S1: Gene Ontology (GO) enrichment analysis of RNA-Seq. Only the top 12 enrichment of GO terms from the “biological process” category are listed here. Gene Set Enrichment Analysis was performed on differentially expressed genes ranked by *p* value using clusterProfiler v.3.4.4. 10^6^ permutations were performed to estimate permutation-based enrichment *p* value.

## References

[1] Thompson W. W., Shay D. K., Weintraub E. (2003). Mortality associated with influenza and respiratory syncytial virus in the United States. *JAMA*.

[2] Thompson W. W., Shay D. K., Weintraub E. (2004). Influenza-associated hospitalizations in the United States. *JAMA*.

[3] Kato H., Sato S., Yoneyama M. (2005). Cell type-specific involvement of RIG-I in antiviral response. *Immunity*.

[4] Kawai T., Akira S. (2011). Toll-like receptors and their crosstalk with other innate receptors in infection and immunity. *Immunity*.

[5] Guillot L., le Goffic R., Bloch S. (2005). Involvement of Toll-like receptor 3 in the immune response of lung epithelial cells to double-stranded RNA and influenza A virus. *Journal of Biological Chemistry*.

[6] Lund J. M., Alexopoulou L., Sato A. (2004). Recognition of single-stranded RNA viruses by Toll-like receptor 7. *Proceedings of the National Academy of Sciences of the United States of America*.

[7] Thomas P. G., Dash P., Aldridge J. R. (2009). The intracellular sensor NLRP3 mediates key innate and healing responses to influenza A virus via the regulation of caspase-1. *Immunity*.

[8] Allen I. C., Scull M. A., Moore C. B. (2009). The NLRP3 inflammasome mediates in vivo innate immunity to influenza A virus through recognition of viral RNA. *Immunity*.

[9] Sabbah A., Chang T. H., Harnack R. (2009). Activation of innate immune antiviral responses by Nod2. *Nature Immunology*.

[10] Michael P., Brabant D., Bleiblo F. (2013). Influenza A induced cellular signal transduction pathways. *J Thorac Dis*.

[11] Kobasa D., Jones S. M., Shinya K. (2007). Aberrant innate immune response in lethal infection of macaques with the 1918 influenza virus. *Nature*.

[12] Kash J. C., Tumpey T. M., Proll S. C. (2006). Genomic analysis of increased host immune and cell death responses induced by 1918 influenza virus. *Nature*.

[13] Baskin C. R., Bielefeldt-Ohmann H., Tumpey T. M. (2009). Early and sustained innate immune response defines pathology and death in nonhuman primates infected by highly pathogenic influenza virus. *Proceedings of the National Academy of Sciences of the United States of America*.

[14] Kumar H., Kawai T., Kato H. (2006). Essential role of IPS-1 in innate immune responses against RNA viruses. *Journal of Experimental Medicine*.

[15] Kato H., Takeuchi O., Sato S. (2006). Differential roles of MDA5 and RIG-I helicases in the recognition of RNA viruses. *Nature*.

[16] Killip M. J., Fodor E., Randall R. E. (2015). Influenza virus activation of the interferon system. *Virus Research*.

[17] Wang X., Wu W., Zhang W. (2017). RIG-I overexpression decreases mortality of cigarette smoke exposed mice during influenza A virus infection. *Respiratory Research*.

[18] Goffic R. L., Balloy V., Lagranderie M. (2006). Detrimental contribution of the Toll-like receptor (TLR)3 to influenza A virus–induced acute pneumonia. *PLoS Pathogens*.

[19] Coulombe F., Fiola S., Akira S., Cormier Y., Gosselin J. (2012). Muramyl dipeptide induces NOD2-dependent Ly6C^high^ monocyte recruitment to the lungs and protects against influenza virus infection. *PLoS One*.

[20] Jewell N. A., Cline T., Mertz S. E. (2010). Lambda interferon is the predominant interferon induced by influenza A virus infection in vivo. *Journal of Virology*.

[21] Weber M., Sediri H., Felgenhauer U. (2015). Influenza virus adaptation PB2-627K modulates nucleocapsid inhibition by the pathogen sensor RIG-I. *Cell Host & Microbe*.

[22] Wang Y., Zhang H. X., Sun Y. P. (2007). *Rig*-I^−/−^ mice develop colitis associated with downregulation of G*α*i2. *Cell Research*.

[23] Errett J. S., Suthar M. S., McMillan A., Diamond M. S., Gale M. (2013). The essential, nonredundant roles of RIG-I and MDA5 in detecting and controlling West Nile virus infection. *Journal of Virology*.

[24] Stegemann-Koniszewski S., Gereke M., Orrskog S. (2013). TLR7 contributes to the rapid progression but not to the overall fatal outcome of secondary pneumococcal disease following influenza A virus infection. *Journal of Innate Immunity*.

[25] Koyama S., Ishii K. J., Kumar H. (2007). Differential role of TLR- and RLR-signaling in the immune responses to influenza A virus infection and vaccination. *The Journal of Immunology*.

[26] Wu W., Zhang W., Duggan E. S., Booth J. L., Zou M. H., Metcalf J. P. (2015). RIG-I and TLR3 are both required for maximum interferon induction by influenza virus in human lung alveolar epithelial cells. *Virology*.

[27] Morosky S. A., Zhu J., Mukherjee A., Sarkar S. N., Coyne C. B. (2011). Retinoic acid-induced gene-I (RIG-I) associates with nucleotide-binding oligomerization domain-2 (NOD2) to negatively regulate inflammatory signaling. *Journal of Biological Chemistry*.

[28] Benitez A. A., Panis M., Xue J. (2015). In vivo RNAi screening identifies MDA5 as a significant contributor to the cellular defense against influenza A virus. *Cell Reports*.

[29] Zhang H., Air G. M. (1994). Expression of functional influenza virus A polymerase proteins and template from cloned cDNAS in recombinant vaccinia virus infected cells. *Biochemical and Biophysical Research Communications*.

[30] Mishra A., Guo Y., Zhang L. (2016). A critical role for P2X_7_ receptor–induced VCAM-1 shedding and neutrophil infiltration during acute lung injury. *The Journal of Immunology*.

