# Supplementary figures and images for: RIG-I Signaling via MAVS Is Dispensable for Survival in Lethal Influenza Infection In Vivo
Source: Mediators Inflamm. 2018 Nov 8;2018:6808934. doi: 10.1155/2018/6808934 (PMC6250004; doi:10.1155/2018/6808934)

**
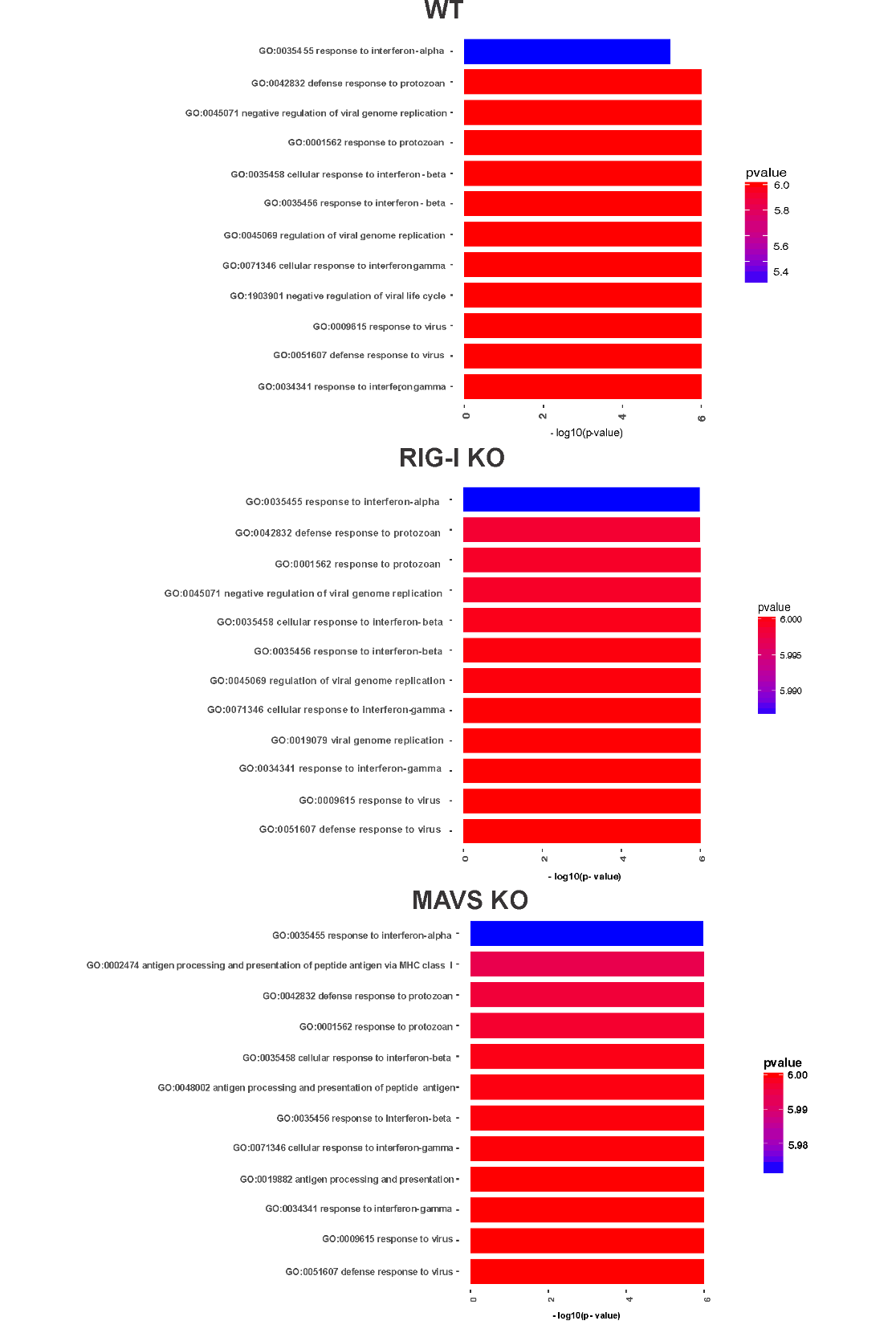
**

Supplement: Supplementary Materials — Figure S1: Gene Ontology (GO) enrichment analysis of RNA-Seq. Only the top 12 enrichment of GO terms from the “biological process” category are listed here. Gene Set Enrichment Analysis was performed on differentially expressed genes ranked by p value using clusterProfiler v.3.4.4. 106 permutations were performed to estimate permutation-based enrichment p value. [file 6808934.f1.docx]
